# Factors Influencing Paternal Attachment Among Preterm Infants in an Urban Neonatal Intensive Care Unit

**DOI:** 10.7759/cureus.8476

**Published:** 2020-06-06

**Authors:** Rickey Taing, Ovidiu Galescu, Lawrence Noble, Ivan L Hand

**Affiliations:** 1 Neonatology, Kings County Hospital, Brooklyn, USA; 2 Pediatric Endocrinology, Federal Drug Administration, Silver Spring, USA; 3 Pediatrics and Neonatology, Icahn School of Medicine at Mount Sinai, Queens Hospital Center, Elmhurst, USA; 4 Neonatal Medicine, Kings County Hospital/State University of New York Downstate School of Medicine, Brooklyn, USA

**Keywords:** preterm, paternal, attachment, neonatal intensive care unit, infant

## Abstract

The aim of this study is to elucidate factors that may influence paternal attachment to preterm infants in an urban hospital setting. Fathers of preterm infants admitted to a level III neonatal intensive care unit (NICU) were eligible for this study. The Paternal Postnatal Attachment Scale (PPAS) is a questionnaire that invokes paternal attachment in five domains: patience, tolerance, pleasure, affection and pride. Clinical and demographic data were collected along with the PPAS to identify factors influencing paternal attachment. Infants studied were 28.1 ± 3.3 weeks gestational age with a birth weight of 1,070 ± 70 grams. Paternal age was 29.2 ± 6.6 years. Infants of fathers who scored in the lowest quartile of attachment were significantly smaller (756 ± 17 grams vs 1,210 ± 76 grams, p = 0.03) and more immature (26.4 ± 1.9 weeks vs 28.8 ± 3.5 weeks, p = 0.04) than infants of fathers with higher attachment scores. Subscores of patience and tolerance (p = 0.05) and pleasure in interaction (p = 0.01) were also significantly lower although there was no significant difference in subscores of affection and pride (p = 0.36). There were no significant differences between attachment scores for paternal age, educational level, marital status, number of children or breastfeeding status. Paternal attachment scores appear to be dependent on infant factors, such as birth weight and gestational age, rather than paternal or demographic factors.

## Introduction

The birth of a preterm infant and ensuing hospitalization in the neonatal intensive care unit (NICU) is a stressful experience for families, with the greatest stress affecting the families of the smallest, sickest infants [1-3]. Although initially an anxiety provoking situation, it has been shown that fathers who have early contact with their infants have reduced anxiety. The sooner fathers are granted the permission to hold their infant, the sooner bonding and attachment is established [4,5]. Current research suggests that paternal attachment influences infant’s sociability and development. On a cognitive scale, those infants with fathers who are engaged in their infants and demonstrate positive perceptions outperform the infants of fathers with more negative feelings. Children of disengaged fathers have also been shown to have an increased incidence of early behavioral problems [6,7]. Thus, assessment and facilitation of early paternal attachment may influence later child behavior and academic achievement.

A great number of psychological studies have been conducted examining early maternal attachment; however, there is a paucity of research examining the paternal role and contribution of attachment [8,9]. The focus of this study is to examine variables that impact developing paternal attachment and bonding.

## Materials and methods

A survey was implemented to evaluate factors that influence paternal attachment and measure fathers’ levels of attachment with their preterm neonates in NICU setting.

Fathers of preterm neonates less than 37 weeks' gestational age and admitted to the NICU were recruited over a six-month period between July and December 2012 at Kings County Hospital Center, a level III NICU in Brooklyn, NY. All preterm infants with a length of stay greater than seven days were eligible for the study. The study sample was comprised of a convenience sample of 27 infants when the researcher (R. T.) was available. Fathers who agreed to be part of the study were given the Paternal Postnatal Attachment Scale (PPAS) questionnaire to assess attachment when their infants were between 7 and 14 days old. The PPAS is a questionnaire that invokes paternal emotions and thoughts in relation to their contact with their neonates and defines three positive attachment factors: patience and tolerance, pleasure in interaction, affection and pride [10]. The PPAS was designed for use during the first year of life and has shown construct validity and reliability. The PPAS allows assessment of father-infant attachment via self-reporting in a clinical setting. The PPAS was administered by one investigator (R. T.) to all study participants over the course of 15-20 minutes in the NICU. Demographic information of the fathers and medical information of the infants were collected and analyzed. The study was reviewed and approved by the Institutional Review Board at State University of New York Downstate School of Medicine.

Data analysis

T tests were used to compare demographic and clinical variables. Analyses of covariance (ANCOVAs) were run for each individual subscore and the total score with paternal factors of age, education, marital status as well as infant factors, including gestational age and birth weight both individually and combined. All analyses were performed using SPSS Statistics 22.0 (Armonk, NY: IBM Corp) using a p level of <0.05 for statistical significance.

## Results

A total of 27 fathers of preterm infants were eligible and available for this study and 23 fathers agreed to participate in the questionnaire. The average age of the fathers was 29.2 ± 6.6 years. All fathers were African-American as reflects the population of this inner city NICU. The average gestational age was 28.1 ± 3.3 weeks with an average birth weight of 1.07 ± 0.67 kg. There were no statistically significant differences in paternal attachment scores for demographic factors, including paternal age, educational level, marital status, number of children or breastfeeding status.

The data yielded from the paternal attachment questionnaire are presented in Table 1. The total score on the PPAS is the sum of three subscores: patience and tolerance, pleasure in interaction, and affection and pride. Reliability analysis was done using SPSS 22 for all three subscores and the total score resulting in an acceptable Cronbach’s alpha of 0.76, thus demonstrating that the scores are similar and related (internally consistent), but each contributing some unique information as well to the overall model.

**Table 1:** Paternal Postnatal Attachment Scale: total and subscores *p < 0.001. SD, standard deviation.

	Mean ± SD	Range	Median	Mode	Skewness	R	First Quartile
Total score	76.6 ± 5.1	62.5-83	76.9	83	-1.3	-	75.5
Patience and tolerance	29.6 ± 3.2	19.9-33	30.6	32	-1.5	0.82*	30.3
Pleasure in interaction	31.7 ± 2.7	25.6-35	32.6	33.6	-0.7	0.77*	14.7
Affection and pride	15.3 ± 1.0	12.6-16	16	16	-1.5	0.35	14.7

Regression analyses between total score and the patience and tolerance subscore showed an R value of 0.82 (p < 0.001). Similarly, the regression analyses of total score and pleasure in interaction had an R value of 0.77 (p < 0.001). The affection and pride subscore had the lowest R value of 0.35, which did not achieve statistical significance. For each of the scores, the first quartile upper limit was identified and a comparison was made between fathers scoring in the lowest quartile (first) and fathers with total scores greater than the first quartile.

Infants with fathers who scored in the lowest quartile had significantly lower birth weights (0.75 ± 0.17 vs 1.2 ± 0.7 kg, p = 0.03) and significantly lower gestational age (26.4 ± 1.9 vs 28.8 ± 3.5 weeks, p = 0.04) (Table 2).

**Table 2:** Comparison of factors with total attachment scores below and above first quartile

	Total score below first quartile	Total score above first quartile	P value
Birth weight (kg)	0.75 ± 0.17	1.2 ± 0.7	0.03
Gestational age (weeks)	26.4 ± 1.9	28.8 ± 3.5	0.04
Paternal age (years)	30.7 ± 2.7	28.6 ± 7.8	0.33
Patience and tolerance	26.9 ± 4	30.7 ± 1.9	0.05
Pleasure in interaction	29.4 ± 2.6	32.6 ± 2.1	0.01
Affection and pride	15 ± 1.15	15.4 ± 0.9	0.36
Total score	71.3 ± 5.5	78.9 ± 2.7	-

In addition, analysis of the subscores demonstrated that fathers who scored in the lowest quartile of attachment scored significantly lower in the categories of patience and tolerance (26.9 ± 4 vs 30.7 ± 1.9, p = 0.05) and pleasure in interaction (29.4 ± 2.6 vs 32.6 ± 2.1, p = 0.01) than fathers with total scores greater than the first quartile. There was no significant association with the factor, affection and pride (15 ± 1.15 vs 15.4 ± 0.9, p = 0.36).

## Discussion

The purpose of our study was to identify factors that may influence paternal attachment with preterm neonates. Bowlby has described attachment as “lasting psychological connectedness between human beings” [11]. It has been suggested that human paternal attachment may begin as early as the second trimester [12-14].

Paternal attachment is a critical component of well-being in the developing infant. Limited or obstructed paternal involvement has been found to have a negative impact on birth outcome and low birth weight, whereas infants who experience positive perceptions from their father demonstrate improvement in weight gain during their hospitalization [15-18].

The PPAS was used as an instrument to assess attachment [10]. It has previously been validated in fathers of infants three, six and twelve months of age. To our knowledge, this is the first study of paternal attachment with preterm infants in a neonatal intensive care unit.

In our study, fathers showed no significant demographic characteristics that influenced their attachment with their preterm infant. This included paternal age, education, marital status, previous infants and breastfeeding status. We did see a significant difference in attachment scores in fathers of the smallest infants in our NICU. Fathers with attachment scores in the lowest quartile had significantly smaller and more immature infants than those scoring above the first quartile. Low gestational age and low birth weight have been identified as factors that influence paternal perception of the infant [19]. We have found that feelings of attachment are affected by these same factors.

The subgroup of fathers scoring lowest in global attachment scores demonstrated lower patience and tolerance and pleasure in interaction scores than the fathers in the top three quartiles. This correlated strongly with their total attachment scores with R values of 0.82 and 0.77, respectively. There was no significant difference between affection and pride scores between the lowest quartile and the remainder of fathers, with a p value of 0.36. Patience and tolerance as well as pleasure in interaction are interactive factors directly influenced by responding to the infant. Affection and pride appear to be more stable among men and may represent an enduring cognition towards the infant, less dependent on interaction.

One limitation of this study is the fact that the paternal attachment questionnaire has not been validated in a NICU population, only in a cohort of six-month-old infants. It was used because there is a lack of paternal attachment instruments available and none have been used in the NICU setting. We feel that is a useful tool and successfully distinguished “low” attachment from “high” attachment fathers. Another limitation is our relatively small sample size in a single urban NICU. Our patient population was exclusively African-American; thus, our results may not be widely generalizable. Father-infant interaction is highly variable in different cultures, populations and age groups [20,21].

Paternal satisfaction has been linked to inclusive interactions with staff with clear explanations and a perception of quality care [22,23]. Receiving explanations concerning the infant’s health status has been shown to allay fears and help with the bonding process. The most immature infants are the ones with the least positive paternal perceptions and incite the highest levels of anxiety in their parents. Reduction in parental anxiety will permit earlier interaction, which may improve attachment [24]. Anxiety reduction can be achieved through the early introduction of the father to the NICU, its equipment and its staff [25,26]. This could be achieved through interventions, such as involvement in daily care, participation in rounds and paternal skin to skin care [19]. Flacking et al. demonstrated that parents felt more engaged with the newborns if they provided “normal” care, such as bathing, changing diapers and putting on clothing [27]. Any delay in permitting parents to have contact with their infant may disrupt the normal attachment process [28].

## Conclusions

Paternal attachment measures were lowest in fathers of the most immature infants. There was no influence of demographic characteristics on paternal attachment in this cohort. We speculate that better support of the father-infant interaction may increase attachment through more positive paternal feelings. Further prospective studies to investigate the effect of interventions geared to increase the fathers’ interaction with their infants are warranted to improve attachment and foster child development.
